# Ceramide Transfer Protein Deficiency Compromises Organelle Function and Leads to Senescence in Primary Cells

**DOI:** 10.1371/journal.pone.0092142

**Published:** 2014-03-18

**Authors:** Raghavendra Pralhada Rao, Luana Scheffer, Sargur M. Srideshikan, Velayoudame Parthibane, Teresa Kosakowska-Cholody, M. Athar Masood, Kunio Nagashima, Prabhakar Gudla, Stephen Lockett, Usha Acharya, Jairaj K. Acharya

**Affiliations:** 1 Laboratory of Cell and Developmental Signaling, National Cancer Institute, Frederick, Maryland, United States of America; 2 Laboratory of Proteomics and Analytical Technologies, Frederick National Laboratory for Cancer Research, Frederick, Maryland, United States of America; 3 Electron Microscopy Laboratory, Frederick National Laboratory for Cancer Research, Frederick, Maryland, United States of America; 4 Optical Microscopy and Analysis Laboratory, Frederick National Laboratory for Cancer Research, Frederick, Maryland, United States of America; 5 Program in Gene Function and Expression, University of Massachusetts Medical School, Worcester, Massachusetts, United States of America; University of Dayton, United States of America

## Abstract

Ceramide transfer protein (CERT) transfers ceramide from the endoplasmic reticulum (ER) to the Golgi complex. Its deficiency in mouse leads to embryonic death at E11.5. CERT deficient embryos die from cardiac failure due to defective organogenesis, but not due to ceramide induced apoptotic or necrotic cell death. In the current study we examined the effect of CERT deficiency in a primary cell line, namely, mouse embryonic fibroblasts (MEFs). We show that in MEFs, unlike in mutant embryos, lack of CERT does not lead to increased ceramide but causes an accumulation of hexosylceramides. Nevertheless, the defects due to defective sphingolipid metabolism that ensue, when ceramide fails to be trafficked from ER to the Golgi complex, compromise the viability of the cell. Therefore, MEFs display an incipient ER stress. While we observe that ceramide trafficking from ER to the Golgi complex is compromised, the forward transport of VSVG-GFP protein is unhindered from ER to Golgi complex to the plasma membrane. However, retrograde trafficking of the plasma membrane-associated cholera toxin B to the Golgi complex is reduced. The dysregulated sphingolipid metabolism also leads to increased mitochondrial hexosylceramide. The mitochondrial functions are also compromised in mutant MEFs since they have reduced ATP levels, have increased reactive oxygen species, and show increased glutathione reductase activity. Live-cell imaging shows that the mutant mitochondria exhibit reduced fission and fusion events. The mitochondrial dysfunction leads to an increased mitophagy in the CERT mutant MEFs. The compromised organelle function compromise cell viability and results in premature senescence of these MEFs.

## Introduction

Sphingolipids are integral to most eukaryotic membranes and intermediates of the sphingolipid metabolic pathway are regulators of various cellular processes such as cell division, differentiation, and cell death [1]. Ceramide, a branch point metabolite in the pathway, is perhaps best known as a pro-apoptotic molecule [2], [3]. However, it plays a fundamental role as a substrate during biosynthesis of a number of important sphingolipid molecules. Ceramide is generated in the endoplasmic reticulum (ER) by a well-conserved *de novo* biosynthetic route. Ceramide transfer protein (CERT) is responsible for the active transport of majority of the ceramide from ER to the Golgi complex where ceramide is converted to sphingomyelin and other complex sphingolipids [4]. CERT is a cytosolic protein that interacts with both ER and Golgi components to facilitate ceramide transfer [5], [6], [7], [8], [9].

*Drosophila* CERT is essential for maintaining the physical characteristics of the plasma membrane [10]. In mammals, CERT function is essential for embryogenesis [11]. CERT null embryos died from cardiac failure with grossly incompetent heart. Increased ceramide was found to be the cause of the defect in the mutant embryos. In the current study, we investigated the consequence of loss of CERT function in a primary cell namely, the mouse embryonic fibroblasts (MEFs). The loss of CERT in primary MEFs does not result in the accumulation of ceramide either in the ER or mitochondria. Instead, there is upregulation of hexosylceramides. We show that the *de novo* biosynthesis of sphingolipids leads to increased levels of hexosylceramide, probably channeling the ceramide that accumulates in the ER, to this product. Despite this effort by the cells to mitigate the toxic effects of ceramide, several organelles are compromised in the mutant MEFs. The cells also exhibit increased levels of autophagy, specifically mitophagy. Ultimately, the chronic stress due to organelle failure culminates in cellular senescence.

## Materials and Methods

### Ethics Statement

The research article meets all applicable standards for the ethics of experimentation and research integrity. This study was carried out in strict accordance with the recommendations in the Guide for the Care and Use of Laboratory Animals of the National Institutes of Health. The protocol was approved by the Animal Care and Usage Committee of NCI-Frederick (NIH) (Animal Study Proposal: 11- 073).

### Isolation of Primary Mouse Embryonic Fibroblasts (MEFs)

CERT deficient mice (*Cert^gt/gt^*) used in this study were generated as described previously [11]. Wild type siblings were used as controls (*Cert^+/+^*). Timed mating was set up between heterozygous mice. E10.5 dpc (day post coitum) embryos were separated from the yolk sac, washed twice with PBS, pH 7.2, and trypsinized for 10 min. The cells were disaggregated by pipetting several times and suspended in 1 ml of pre-warmed (37°C) DMEM supplemented with 10% FBS. The suspension was allowed to stand for 2–3 min at room temperature, and visible clumps were removed. It was further centrifuged at 500 *g* for 3 min, and the cell pellet was suspended in MEF media (DMEM +10% FBS) and plated on a 6-well plate. The plates were incubated at 37°C with 3% oxygen and 5% carbon dioxide as described previously. DNA isolated from yolk sacs was used for genotyping as described previously [11].

### Sphingolipid Analysis

Lipids extracts were prepared from MEFs in passage number 3 and sphingolipids were estimated as described previously [10], [11]. The individual sphingolipid species of a given class was combined to calculate the total amount of each class of sphingolipid and normalized to one hundred percent for the *Cert^+/+^* and compared with the correspondingly calculated mutant sphingolipid.

### Metabolic Radiolabeling of Sphingolipids

L-[U-^14^C]-Serine (5.3 GBq/mmol) was from Perkin Elmer. Thin layer chromatography (TLC) plates (aluminum sheets of silica gel 60) were from EMD Millipore. For metabolic labeling 1×10^6^ MEF cells in the culture medium were labeled with 10 µcurie of [^14^C]-Serine for 24 hours. Equal number of cells were harvested, total cellular lipids extracted and the lipids were separated by TLC using methyl acetate/n-propanol/chloroform/methanol/0.25% potassium chloride (25/25/25/10/9; v/v) solvent system. After resolution, the TLC plates were exposed to autoradiography for 48 hours. The R_f_ values for ceramide, hexosylceramide, sphingomyelin, phosphatidylethanolamine and phosphatidylserine were 0.96, 0.73, 0.08, 0.38 and 0.21, respectively.

### VSVG-GFP transport assay

VSVG-GFP transport assays were performed as described before with some modifications [12], [13]. Briefly, MEFs were plated on 35 mm petridish with 14 mm microwell. The following day, cells were transfected with VSVG-GFP vector using lipofectamine and were incubated at 40°C for a further 20 h. All subsequent incubations were done in the presence of 50 μg/ml cycloheximide (Sigma) to halt protein synthesis. For time t = 0, cells were rinsed in ice-cold PBS before fixation. Remaining cells were returned to an incubator at 32°C for the indicated time (30, 60 and 180 min). After incubation, cells were rinsed in ice-cold PBS and fixed in 4% paraformaldehyde. For treatment with WGA, cells were incubated with 0.01 mg/ml WGA-Texas red on ice for 10 min. For PDI and GM130 staining, cells were permeabilized with 0.2% Triton X-100 and stained using anti-GM130 and anti PDI, followed by Alexa 647 anti mouse and anti-rabbit secondary antibody respectively. Cells were imaged using a Zeiss LCI510 confocal microscope.

### Cell proliferation assay

Cell proliferation was quantitated by MTT assay. MEFs were plated at 500 cells/well in 96 well plates and were allowed to attach for 12 hours. At different time points MTT was added and plates were incubated at 37°C for 4 hours after which the reaction was arrested by adding the stop solution. The plates were incubated at 25°C overnight and absorbance was recorded at 550 nm.

### Cell passage

5×10^4^ freshly isolated MEFs were plated in 35 mm dish and were incubated at 37°C with 3% oxygen and 5% carbon dioxide for 3 days after which they were passaged at same density. Cells were subjected to different passages following this protocol and fold increase in the cell count during each passage was calculated. Fold change in 72 hours was used as an index of mitosis.

### Senescence assay

25×10^4^ cells were plated in 35 mm well, incubated for 18 hours at 37°C with 3% oxygen and 5% carbon dioxide. Senescence assay was performed using the β-galactosidase assay kit from cell signaling following manufacturers protocol. This assay is specific for senescence induced β-galactosidase and briefly, the adherent cells were washed twice with ice cold PBS and once with the assay buffer provided in the kit. The cells were fixed in the fixative for 10 minutes and again washed in the assay buffer followed by incubation with the β-galactoside staining solution provided in the kit, for 24 hours at 37°C in dry incubator (with no carbon dioxide). Finally cells were washed in PBS and visualized under microscope.

### Electron microscopy

For EM analysis cells were washed in PBS and fixed in cacodylate buffer containing 2% glutaraldehyde and stored at 4°C. Samples were then sectioned and analyzed by transmission electron microscopy [10], [11].

### Western blots

The cells were collected by trypsinization and washed twice in PBS. They were lysed in RIPA buffer (supplemented with protease inhibitors) and 15–20 μg of protein was separated on 4–12% bis-tris gel. The protein bands were transferred to PVDF membrane and incubated with blocking solution (5% BSA) 1–2 hours. Following this the membranes were probed with appropriate primary and secondary antibodies and detected by chemiluminiscence.

### Immunocytochemistry

For Golgi staining, adherent cells were grown on glass bottom dishes coated with poly-lysine (MatTeK, Ashland, MA), and were fixed with 4% paraformaldehyde for 30 min at room temperature. After Triton X-100 permeabilization, cells were blocked for 1 hour with 5% horse serum in PBS and then incubated with anti-GM130 antibody (610822; BD Transduction Laboratories) for 90 minutes and secondary antibody (Alexa 488 goat anti- mouse IgG) for 1 hour. Cells were extensively washed with PBS after incubations with primary and secondary antibodies and were counterstained with DAPI for nuclei and mounted with Vectashield. Cells stained with secondary antibody alone were used as negative controls.

Fluorescence microscopy was performed using a fluorescence microscope (LSM 510 META; Zeiss) equipped with 63x/1.4NA oil immersion objective. Images were acquired with the use of proprietary Zeiss software package.

For LC3B staining cells were grown on 35 mm glass bottom dish, washed with PBS, fixed in 4% paraformaldehyde solution for 10 min at room temperature, and permeabilized with 0.2% Triton +1% BSA in PBS for 10 min at room temperature. Immunofluorescence analysis was performed using anti LC3B antibody (Cell Signaling) and Alexa Fluor 488-conjugated secondary antibody (Invitrogen). Confocal laser-scanning microscopic analysis was performed using a LCI510 (Zeiss). Only cells with more than 5 punctae were considered positive for the analysis. 200 cells were counted in each and the data represents average of at least 2 KO MEF lines.

Live cell trafficking of ceramide from ER to the Golgi complex was monitored by incubating the MEFs with 5 μM DMB-C5-Cer (Bodipy-C5-ceramide) for 30 min at 4°C. Cells were then washed and further kept at 37°C for another 20 min.

### Fluorescence recovery after photobleaching

Cells were incubated with 1 μg/ml AF488 Ctx B (invitrogen) for 30 min in cold (4°C), washed and further incubated for 60 min at 37°C. CTxB was internalized and accumulated in a perinuclear region corresponding to Golgi apparatus. The whole Golgi area was emptied of fluorescence by bleaching using a high-energy laser scan of the confocal microscope. Low intensity illumination was then used to monitor recovery of fluorescence into the bleached area and images were acquired at intervals of 2 min, until level of fluorescence remained constant between consecutive scans.

### Image Acquisition

To quantify the mitochondrial dynamics, time-lapse 3-D stacks for both cell types were acquired using Zeiss LSM 510 confocal microscope (Carl Zeiss Gmbh, Zena, Germany) equipped with environmental chamber, 63x/1.4NA oil immersion objective and a diode laser (excitation wavelength of 561 nm). The acquisition parameters were as followed: approx. 1% laser power for excitation of the mitoTracker Red CMX; zoom of 1.5 with line averaging of 2; pinhole of 1 Airy unit, X-Y pixel size of 0.09 μm, z-spacing of 1 μm, and a time step of 109 s. All images were 8-bit and covered an area of 1024×1024 pixels in the X-Y plane (∼84×84 μm), 4-6 z-sections (∼4–6 μm), and at least 8 time intervals (i.e., the experiment was ≥16 min).

### Image Analysis

First, a best-in-focus plane along the z-axis was visually selected from the 3-D time series stack and then 3–4 regions of interest (ROIs) were selected along the time-series from the perinuclear region of a cell.

In the WT, we used a semi-automatic segmentation, Simple Neurite Tracer plug-in (SNT) from FIJI, to identify mitochondria [14]. SNT uses a Hessian-based analysis of the image to identify the best (mathematically) path (centerline) between user-selected start and end points. The result of this semi-automatic operation is typically a single-pixel wide segmentation representing the underlying tube-like structure (e.g., mitochondria in WT cells). This process was repeated for all time steps. The single-pixel wide tube-like representation was then saved as a binary TIFF stack (0 represent background where as 1 represents the segmented mitochondria).

The mitochondria in mutant cells were automatically segmented using an adaptive local thresholding technique. Briefly, this method entails examining the mean of the minimum and maximum intensity values of the local neighborhood (radius  = 15 pixels) at each pixel in the image to estimate a local threshold value [15]. An implementation of this method was readily available as plugin in FIJI, MidGrey method in Auto Local Threshold [14]. A medial-axis transform (MAT) was applied to extract the centerline representation of mitochondria. The resulting single-pixel wide centerlines were saved as a binary TIFF stack as above.

Note that several techniques were tested to unify the segmentation process in both cell types; however, none yielded satisfactory results. This is not surprising because of the vast difference in mitochondria morphology in WT and mutants.

### Tracking

To quantify mitochondrial dynamics, the centerline representing mitochondria had to be linked in each time step. A robust, automatic tracking algorithm proposed by Crocker *et al* was used in our studies [16]. A MATLAB (Mathworks, Inc., Natick, MA) implementation of their algorithm (*track.m*) was used to automatically link/track the objects in the time-series. The segmented time-series TIFF stacks were preprocessed to conform to the input requirements of their algorithm. This entailed calculating the geometric center (centroid) of single-pixel wide centerline representation of the mitochondria at each time step. Additional parameters, most importantly, the estimate of maximum distance that each mitochondrion can move in a single time step and allowance for mitochondria to reappear after being lost in few time steps, were adjusted accordingly. After the mitochondria were tracked, their length, displacement, and directionality were calculated using an in-house MATLAB algorithm.

### Mitochondrial superoxide generation

MitoSOX 5.0 μM was added to the cells and incubated for 20 min at 37°C in 5% CO_2_ atmosphere. After this period, cells were washed with pre-warmed buffer and counterstained with DAPI. Fluorescent images were taken using an inverted confocal laser scanning microscope (LSM 510 META; Zeiss, Germany) equipped with 63x/1.4NA oil immersion objective at 512×512 pixels. An excitation wavelength of 514 was used to excite MitoSOX dye.

## Results and Discussion

### *Cert^gt/gt^* cells undergo senescence

*Cert^gt/gt^* primary mouse embryonic fibroblasts (MEFs) could only be derived from E9.5 to E10.5 embryos and cultured *ex vivo* under lower oxygen conditions (3% oxygen) as compared to routinely used atmospheric oxygen levels (22% oxygen). The lower oxygen conditions are thought to reflect the physiological conditions of oxygen availability for many of the tissue cells [17]. Even under 3% oxygen, when control MEFs can easily grow past 20 passages most *Cert^gt/gt^* MEFs stop growing by passage 5 and a great majority of them cease to divide by passage 10.

**Figure 1:**
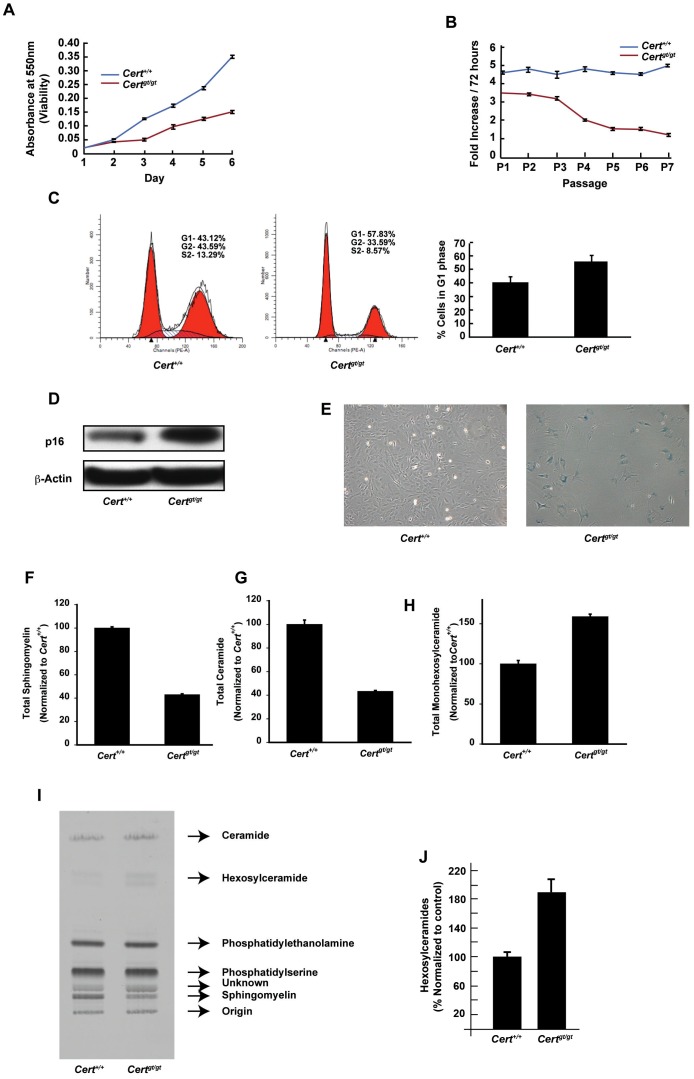
*Cert^gt/gt^* MEFs undergo senescence and have dysregulated sphingolipid metabolism. (A) Cell viability assay of *Cert^gt/gt^* and *Cert^+/+^* MEFs by MTT assay. (B) Mitotic index of *Cert^gt/gt^ Cert^+/+^* MEFs measured over seven passages (C) The MEFs were analyzed by FACS analysis (representative plots on the left) using propidium iodide for staining cells and the percentage of cells in G1 phase measured and plotted (right panel). The actual raw data from a representative experiment along with the multiline plot generated using ModFit LT computer software is shown in the left panels. The red histograms show the percent cells in G1 and G2/M phase while the hatched plot shows the cells in S phase. (D) Upregulation of p16 *Cert^gt/gt^* MEFs. (E) Morphological analysis of late passage cells from control and mutant MEFs. The mutant MEFs show flattened morphology typical of senescent cells. (F) The total sphingomyelin and (G) total ceramide was decreased while total hexosylceramides (H) were increased in the in *Cert^gt/gt^* compared to MEFs derived from *Cert^+/+^* as measured by mass spectrometry. (I) Metabolic labeling with radiolabeled serine showed that the levels of sphingomyelin synthesized by the *de novo* pathway is decreased, while hexosylceramide is increased during the period of pulse chase. (J) The quantification of hexosylceramides from three metabolic labeling experiments is shown. The bands representing hexosylceramides were scraped and counted in a liquid scintillation counter and normalized to the wild type levels.

The proliferation rates of the MEFs were assessed by MTT assay. As shown in Fig. 1A, *Cert^gt/gt^* cells have decreased proliferation rate compared to *Cert^+/+^* cells and stop dividing by passage 6 (Fig. 1B). FACS analysis using propidium iodide stained cells indicated that mutant cells might be arrested in G1-S transition phase (Fig. 1C). In addition to this we found that in these cells, the levels of the senescence marker p16 was increased [18] (Fig. 1D). Most primary *Cert^gt/gt^* MEFs acquired a flattened morphology, characteristic for senescent phenotype (Fig. 1E) and also stained intensely positive for senescence activated β-galactosidase activity.

CERT deficiency is expected to accumulate ceramide in the ER and it is a known pro-apoptotic molecule. Therefore, we decided to investigate the possible link between CERT deficiency and senescence in the primary MEFs.

### CERT deficiency results in increased generation of hexosylceramide

We quantified the steady state levels of sphingolipids in *Cert^+/+^* and *Cert^gt/gt^* MEFs using a combination of normal phase chromatography (NPC), electrospray ionization (ESI) and single reaction monitor (SRM) tandem spectrometry as described previously [10], [11]. We observed that the sphingomyelin levels were relatively low in the *Cert^gt/gt^* as compared to *Cert^+/+^* MEFs (Fig. 1F). The *de novo* pathway generates ceramide in the ER. In the absence of CERT, we expect ceramide accumulation in the mutant cells. Surprisingly, the total ceramide levels were decreased in the mutant MEFs (Fig. 1G). We also found that total monohexosylceramides were increased (by about 50%) in the mutant MEFs compared to the *Cert^+/+^*controls (Fig. 1H and I). To evaluate if hexosylceramide was indeed generated by the *de novo* pathway we employed a pulse chase paradigm and followed the fate of newly synthesized sphingolipids using radiolabeled serine, which condenses with a fatty acyl CoA (generally palmitoyl CoA) in the first committed step of *de novo* biosynthesis and is converted to ceramide. Our results indicate that the *de novo* biosynthetic pathway is compromised in the mutant (Fig. 1I). The amount of sphingomyelin synthesized, from labeled serine, is consistently reduced by about 2.5 fold, in mutant cells. Also, the amounts of hexosylceramides generated are higher by a factor of 2 in *Cert^gt/gt^* MEFs (Fig. 1J). Accordingly, the increased steady state levels of hexosylceramides seen in *Cert^gt/gt^* MEFs are mostly derived from the *de novo* biosynthetic pathway.

### CERT mutant MEFs (*Cert^gt/gt^*) display incipient ER stress

**Figure 2:**
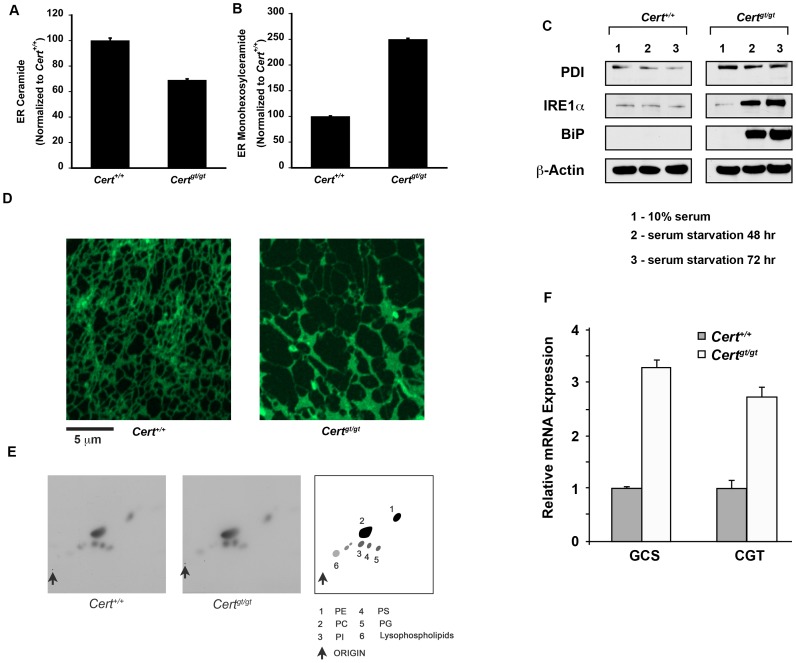
CERT deficiency leads to incipient ER stress in *Cert^gt/gt^* MEFs. (A) The total ER ceramide levels are slightly decreased while (B) hexosylceramide levels are increased in *Cert^gt/gt^* compared to the control *Cert^+/+^* MEFs. (C) Basal PDI levels are increased in the mutant MEFs. Upon serum starvation the levels of IRE1α and BiP are increased in the mutant cells. (D) The ER was labeled with ER tracker green. The ER in the mutant show altered morphology and altered dynamics (see videos S1 and S2). (E). Metabolic labeling for phospholipids were performed using ^32^P-orthophosphoric agent as a substrate. No visible difference in major phospholipids was observed between the wild type control and the mutant cells. (F) qPCR analysis of transcripts for GLUCS and GALCS shows increased transcript levels in P5 *Cert^gt/gt^* compared to the *Cert^+/+^*MEFs.

Since CERT transports ceramide out of the ER to the Golgi complex, we anticipate ceramide levels to be increased in the ER of *Cert^gt/gt^* MEFs. Therefore, we isolated ER by density gradient centrifugation and estimated steady state ceramide and hexosylceramide levels in the ER. Surprisingly, we observe that ceramide levels are decreased in the endoplasmic reticulum in the *Cert^gt/gt^* MEFs (Fig. 2A). It was likely, that in MEFs the increased ceramide was being shunted to hexosylceramide to perhaps mitigate the toxic effects of ceramide. We see a 250% increase in their level (Fig. 2B). To test if the perturbed sphingolipid metabolism has a deleterious effect on the ER, we performed Western blot analysis to compare the steady-state levels of several proteins implicated in the ER stress response and found changes in the levels of BiP, protein disulfide isomerase (PDI) and IRE1α under some conditions. Notably, the levels of PDI were constitutively elevated in the mutant MEFs. IRE1α is a proximal sensor of the ER stress and contributes to monitoring the quality of the proteins synthesized in the ER. PDI and BiP are chaperones in the ER stress pathway [11]. In the presence of serum, *Cert^gt/gt^* MEFs did not show a marked increase in IRE1α and Bip levels. But upon serum starvation for 24–48 hours, there was an increase in the levels of both Bip and IRE1α in *Cert^gt/gt^* compared to *Cert^+/+^* MEFs (Fig. 2C) Finally, we stained the ERs from control and mutant MEFs with ER-tracker green and observed the dynamics of ER. We found a significant change in the rate of reorganization of ER network in the mutant MEFs compared to *Cert^+/+^*control MEFs as observed by time-lapse video of the ER stained ER network over a period of 5 min (Fig. 2D and videos S1 and S2). While noteworthy it is difficult to link this with underlying biochemical defects in the sphingolipid metabolism or ER stress. Alterations in the *de novo* sphingolipid biosynthetic pathway could indirectly affect lipid biosynthetic pathways in the ER by compromising ER's function. Therefore, we evaluated the phospholipid biosynthetic potential of the cells using ^32^P-orthophosphoric acid as substrate and observed synthesis over a period of 24 hours. As can be seen in Fig. 2E there is no deficiency in phospholipid biosynthesis in the in *Cert^gt/gt^* MEFs. Serine labeling that gets incorporated into phosphatidylserine and phosphatidylethanolamine also shows no difference (Fig. 1I).

To evaluate protein trafficking we transfected the cells with VSVG-GFP and monitored its synthesis and transport [12]. At 0 min after induction we observe VSVG-GFP co-localizes with PDI both in the control and the mutant cells (Figure S1). At 30 min after induction we begin to see co-localization with a Golgi marker (GM130) that is pronounced at 60 min and at about 180 min we see plasma membrane localization of VSVG-GFP in both wild type and mutant MEFs.

The conversion of ceramide to hexosylceramide could be facilitated by galactosylceramides synthase (GALCS), an ER resident protein or glucosylceramides synthase (GLUCS) [19]. GLUCS is generally described as an enzyme residing on the cytosolic side of the medial Golgi, but the *Drosophila* enzyme has been shown to localize to the ER and one group has purified it in the mitochondria associated membranes a specialized compartment of the ER [20], [21], [22], [23]. qPCR analyses of P5 MEFs suggest that transcripts for both genes are increased in the mutant MEFs (Fig. 2F). Thus, the ceramide that is not transferred to the Golgi complex in the mutant could be acted by both these enzymes and converted to either galactosylceramides or glucosylceramides. While this mitigates the acute toxic effects of ceramide, and supports basal protein transport or other lipid biosynthetic pathway, the ER is still undergoing incipient ER stress.

### Alterations in Golgi functions in *Cert^gt/gt^* cells

**Figure 3:**
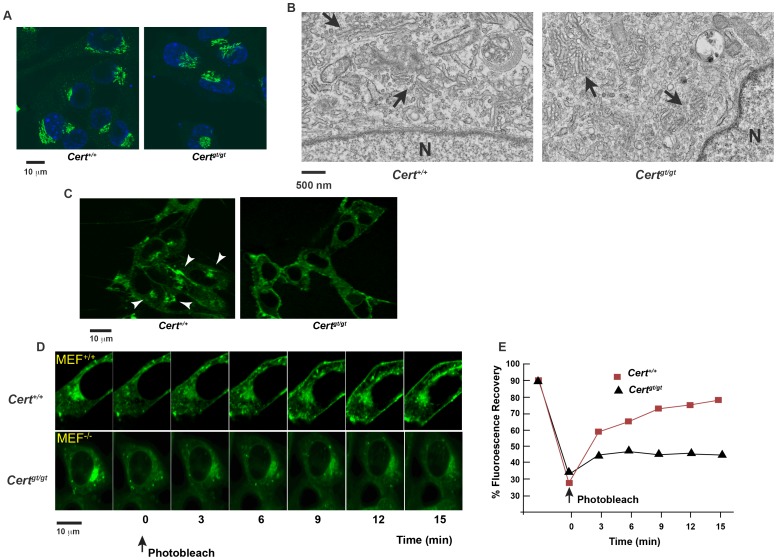
Altered Golgi dynamics in *Cert^gt/gt^* MEFs. (A) The Golgi architecture seems to be normal as evidenced by immunofluorescence staining using a Golgi marker (GM130) and (B) The ultrastructure of Golgi seems apparently normal although we see a slightly more fragmented pattern of Golgi compared to the wild type. N- denotes the nucleus and the arrows indicated the Golgi cisternae. (C) The MEFs were incubated with DMB-C5-Cer as described under materials and methods. While perinuclear concentration of the ceramide is clearly visible in *Cert^+/+^* the *Cert^gt/gt^* show a diffuse staining indicating a lack of transport of ceramide from the ER to the Golgi complex. (D) Recycling of cargo protein cholera toxin B (CTxB, associated with lipid rafts) between the plasma membrane and the Golgi complex is impaired in the mutant MEFs. (E) Quantification of the extent of recovery after photobleaching.

We found the Golgi morphology was similar in *Cert^+/+^* and *Cert^gt/gt^* cells when examined for Golgi marker GM130 (Fig. 3A) or by electron microscopy (Fig. 3B). Since CERT is responsible for the transfer of ceramide from ER to Golgi, we checked whether transfer of ceramide to Golgi is compromised in *Cert^gt/gt^* MEFs by monitoring BODIPY-C5-Cer redistribution in cells [24], [25]. BODIPY-C5-Cer is a lipid fluorescent analog that, when exogenously added in cold (4°C) to the cells and then chased at 37°C for a short period of time (0–5 min), is localized to cytoplasm and ER. After longer chase period, it is redistributed to Golgi apparatus, representing a useful tool to track ceramide movement between the two organelles.

In both *Cert^+/+^* and *Cert^gt/gt^*, BODIPY-C5-Cer was localized to the cytoplasm and ER immediately after incubation at 4°C. However, following 20 minutes of incubation at 37°C, most of the fluorescence accumulated in the perinuclear region representative of the Golgi apparatus only in *Cert^+/+^* cells (Fig. 3C). In contrast, in *Cert^gt/gt^* mutant cells, lack of CERT function led to almost no distribution of the fluorescent lipid between ER and Golgi, indicating the absence of ceramide transport between the two organelles.

CERT function has been implicated in protein trafficking [26]. Surprisingly as described earlier, we did not notice a difference in the ability of *Cert^gt/gt^* cells to synthesize and traffic VSVG-GFP from ER to the Golgi complex or from the latter to the plasma membrane (Figure S1). Thus, at least the trafficking of VSVG-GFP seems to be not compromised in the *Cert^gt/gt^* cells.

We then examined the retrograde transport of Cholera toxin B (CTxB) from the plasma membrane to the Golgi complex. CTxB binds to cell surface GM1which acts as a receptor and CTxB moves along the endocytic compartments to the trans-Golgi network before being transported to the ER [27], [28]. We employed fluorescence recovery after photobleaching (FRAP) to determine the intracellular mobility of fluorescent CTxB, specifically from the plasma membrane to the Golgi complex. FRAP provides the relative extents of fluorescent recovery (mobile fractions), that qualitatively describe the proportion of protein that is able to move between the two organelles. After incubation at 37°C for 60 min, CTxB is trafficked to Golgi both in *Cert^+/+^* and *Cert^gt/gt^* cells. We bleached the whole Golgi compartment and after 15 min the recovery of fluorescence was 50±10% in the mutant, while in the control there was an almost complete recovery of 90±5% (Fig. 3D and E). This indicates that in *Cert^+/+^* CTxB traverses through intracellular compartments efficiently, while in mutant the exchange of cargo between Golgi and PM is impaired in *Cert^gt/gt^* cells.

Thus, deficiency of CERT affects some of the trafficking events related to Golgi such as the trafficking of CTxB while trafficking of a protein such as VSV-G was not severely compromised.

### *Cert^gt/gt^* cells show compromised mitochondrial dynamics and function

Since ER is functionally linked to mitochondria and sphingolipid metabolism in ER of *Cert^gt/gt^* cells is compromised, we evaluated if the structural and functional integrity of mitochondria was compromised and was a contributing factor to the senescence phenotype of these cells.

**Figure 4:**
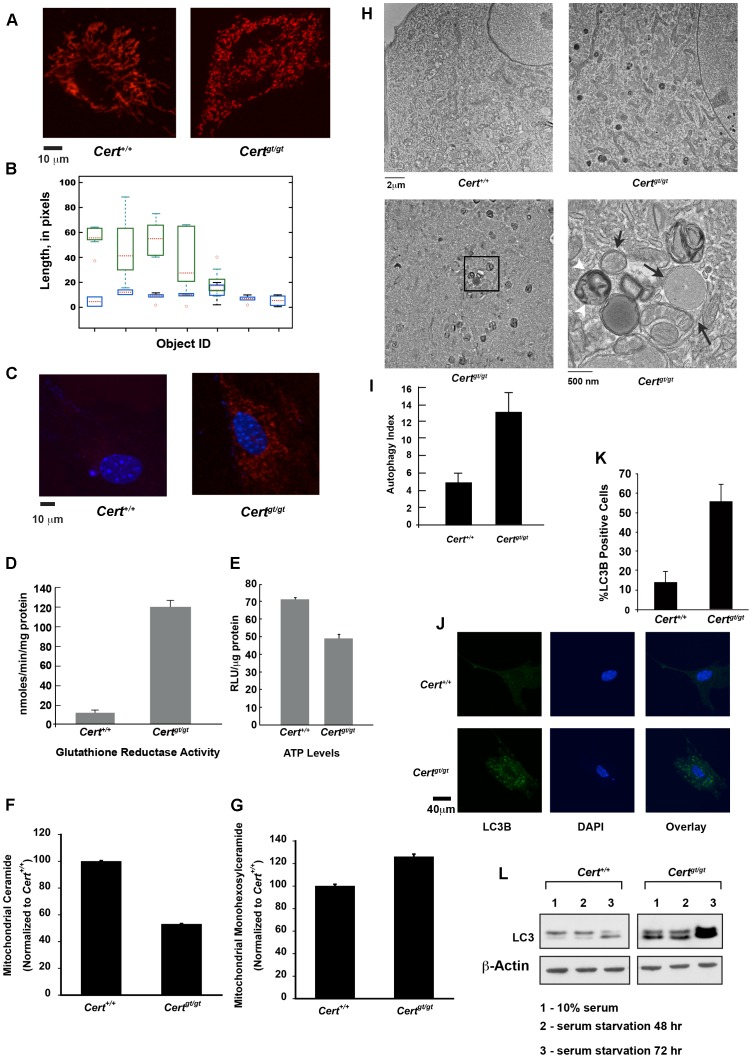
Mitochondrial dysfunction and mitophagy in *Cert^gt/gt^* MEFs. (A) Live cell imaging of mitochondria show that mutant mitochondria appear fragmented and have decreased fusion and fission events (see videos S3 and S4). (B) A measure of pixel variation was used to quantitate the mitochondrial dynamics as described in materials and methods under mitochondria segmentation and tracking. While pixel length change dramatically in the wild type over the period of observation (16 min) the movement of the mutant mitochondria was dramatically reduced *viz*. the length of the mitochondria measured in pixels is changed in wild-type much more than in the mutants (C) The amount of ROS generated was detected using MitoSox reagent and imaged under 514 nm excitation. Increased ROS generation can be observed in *Cert^gt/gt^* MEFs. (D) Glutathione reductase activity is increased while (E) ATP levels are decreased in the mutant cells. (F) Mitochondrial ceramide levels are decreased while (G) the monohexosylceramides are increased in the *Cert^gt/gt^* cells. (H-I) Electron micrographs of passage 6 *Cert^+/+^* MEF (upper left panel) and a passage 5 *Cert^gt/gt^* MEF (upper right panel) shows increased autophagic vacuoles in the latter seen as dark multivesicular structures. A passage 3 *Cert^gt/gt^* MEF showing active mitophagy (lower left panel). The boxed area has been enlarged in the lower right panel showing several mitochondria in various stages of mitophagy. Black arrows show double membrane structure enveloping intact mitochondrion and some in early to mid-stage autophagic degradation. White arrow heads late stage autophagosome. (I) The number of autophagic vacuoles counted from EM images of passage 3 MEFs was counted (n = 15) and the average number was plotted as autophagic index. (J) LC3B staining of *Cert^gt/gt^* and *Cert^+/+^* MEFs. (K) More than 200 cells were counted for each genotype and cells showing more than 5 punctae that were larger and brighter than background staining were considered positive and plotted. (L) Western analysis of LC3 in the control and mutant MEFs.

We stained the live cells with MitoTracker Red CMX, a cell-permeant mitochondrion-selective dye. The mitochondrial morphology in the control *Cert^+/+^* MEFs showed a regular tubular network (Fig. 4A), while the *Cert^gt/gt^* MEFs exhibited a severely damaged mitochondrial network with short and fragmented mitochondria (Fig. 4A). The average size of mitochondrial filament was 5–10 μm in the control, while it was 1–3 μm in the mutant. Mitochondrial dynamics, e.g., motility and displacement, extracted from time-lapse experiments on both cell types showed drastic difference (see videos S3 and S4). In the mutant MEFs the mitochondria were less mobile than in control, with fusion/fission events being practically arrested. Quantization of mitochondrial dynamics showed that in *Cert^+/+^*mitochondria the dynamic range is 100% of their length where as in case of mutant it is only about 15% *i.e*., wild type mitochondrial filaments varied from half the average size to twice as long, whereas the variation was only 15% in the average length in the mutant, during the period of observation (Fig. 4B). These results indicate that the mitochondrial integrity was compromised in the mutant cells.

Mitochondrial integrity is important for tight coupling of electron transport with redox reactions and ATP generation. Structural defects in mitochondria result in electron leak during oxidative phosphorylation that results in the generation of reactive oxygen species and oxidative stress [29], [30], [31]. Since mitochondria seemed compromised in *Cert^gt/gt^* MEFs we evaluated the level of superoxide in the mitochondria. Superoxide anion production was increased in the mutant mitochondria, indicating that the cells were under oxidative stress (Fig. 4C). During oxidative stress antioxidant enzymes are elevated as a defense response. Glutathione reductase is one of the antioxidant enzymes used by cells as a protective mechanism against stress [32]. CERT mutant cells seem to be trying to counteract the oxidative stress by elevating the glutathione reductase activity up to 10 fold (Fig. 4D). Apparently this level of activation seems insufficient to cope with the mitochondrial oxidative stress.

Since mitochondrion is the site of oxidative phosphorylation and a major source of ATP, we hypothesized that damaged mitochondria could be less efficient in ATP production. Therefore, we measured the levels of total ATP in these cells and we found that the mutant mitochondrial ATP level was about 70% relative to the *Cert^+/+^*MEFs (Fig. 4E). Finally, we evaluated whether altered sphingolipid composition was a contributing factor in mitochondrial pathology. Indeed we saw a reduction in relative amounts of ceramide in the mitochondria prepared from *Cert^gt/gt^* MEFs while the levels of monohexosylceramide showed an increase, a trend similar to that observed in ER sphingolipid estimations (Fig. 4F and G). These results strongly suggest that altered sphingolipid metabolism disrupted mitochondrial function even without increased ceramide levels.

### Compromised ER and mitochondrial functions lead to autophagy in *Cert^gt/gt^* cells

One of the defects observed during electron microscopic examination of the late passage (P5–P6) *Cert^gt/gt^* MEFs was the accumulation of multi-membrane vesicular structures in the close proximity of mitochondria (Fig. 4H). Upon careful examination, we found that mutant cells exhibited increased mitophagy Although some amount of autophagy does occur even in wild type primary MEFs there were abnormally high amounts of mitochondria that were undergoing mitophagy in the *Cert^gt/gt^* MEFS. We found both early and late stage autophagosome structures surrounding degrading mitochondria in the mutant (Fig. 4H lower panels). The number of autophagosomes was counted in several fields and defined as autophagy index. The amount of autophagic vesicles in passage 3 cells of *Cert^gt/gt^* was 2.5 fold higher than in the *Cert^+/+^* MEFs (Fig. 4I).

We also established increased incidence of autophagy in the *Cert^gt/gt^* cells by LC3B staining as shown in Fig. 4J [33]. During autophagy, LC3 is lipidated by an ubiquitin-like system involving Atg7 to become associated with autophagic vesicles. Immunofluorescence imaging of *Cert^gt/gt^* showed almost 3–4 fold increase in LC3B staining of cells compared to *Cert^+/+^* cells (Fig. 4K).

We evaluated the mutant cells for biochemical evidence of autophagy. We found an increase in the basal levels of LC3-II in the *Cert^gt/gt^* compared to *Cert^+/+^* cell (Fig. 4L). Serum starvation, a condition that is known to induce autophagy even in wild type cells, caused further increase in the levels of LC3-I and II in mutant cells compared to the wild-type control (Fig. 4L).

Earlier studies from our laboratory demonstrated that at the organismal level CERT function is essential for stress response, aging and organogenesis [10], [11]. Mice lacking functional CERT protein are embryonic lethal and show defects in organogenesis. Embryos showed increase in the intracellular levels of ceramide in the ER and the mitochondria that compromises mitochondrial function and affects cardiogenesis. Ceramide has been shown to be a pro-apoptotic molecule. There are studies, which indicate that endogenous levels of ceramide increase in response to several pro-apoptotic stimuli [35]. In addition to this, *Cert^gt/gt^* MEFs exhibit ER stress which is shown to be one of the triggers for pro apoptotic signaling [36]. Besides, the mitochondria suffer from elevated levels of superoxide and decreased ATP production. An extreme consequence of these events would be cell death mediated through apoptosis. However, preliminary study indicated that *Cert^gt/gt^* MEFs did not undergo apoptosis but were capable of undergoing apoptotic cell death upon exposure to Actinomycin [11]. This has also shown to be the case in the primary MEFs derived from *Cert^gt/gt^* embryos. One can expect that, in the absence of CERT function, ceramide accumulates in the ER and subsequently might move toward mitochondria as we observed in the embryos. Mass spectrometric data and pulse labeling studies show that levels of intracellular sphingolipids are altered in *Cert^gt/gt^* MEFs. However, the mutant MEFs do not show accumulation of ceramide in the ER and mitochondria, rather the levels of hexosylceramides are increased in both organelles. The conversion of ceramide to hexosylceramide could be facilitated by galactosylceramides synthase, an ER resident protein [19]. Upregulation of hexosylceramide could also be catalyzed by glucosylceramides synthase (GCS). GCS is generally described as an enzyme residing on the cytosolic side of the medial Golgi, but the *Drosophila* enzyme has been shown to localize to the ER and one group has purified it in the mitochondria associated membranes a specialized compartment of the ER [20], [21], [22], [23]. Whilst conversion of ceramide to hexosylceramide might mitigate some of the acute toxic effects of the former the cell is still functioning under stress. The slow but continued generation of hexosylceramide could interfere with the structure and function of ER perhaps manifested as incipient ER stress and altered dynamics. Also, the altered ER sphingolipid flux affects mitochondrial structure and function that culminates in mitophagy. One can expect that in *Cert^gt/gt^* MEFs, in the absence of CERT function, ceramide accumulates in the ER and subsequently might move toward mitochondria as we observed in the embryos. Ceramide has been shown to be a pro-apoptotic molecule. There are studies, which indicate that endogenous levels of ceramide increase in response to several pro-apoptotic stimuli [35]. In addition to this, *Cert^gt/gt^* MEFs exhibit ER stress which is shown to be one of the triggers for pro apoptotic signaling [36]. However our studies with mass spectrometric analysis and pulse labeling studies show that although levels of intracellular sphingolipids are altered in *Cert^gt/gt^* MEFs, they do not show accumulation of ceramide in the ER and mitochondria, rather the levels of hexosylceramide are increase in both organelles. Whilst conversion of ceramide to hexosylceramide might mitigate some of the acute toxic effects of the former the conversion cannot correct the abnormal flux of sphingolipids in the absence of CERT. The slow but continued generation of hexosylceramide could interfere with the structure and function of ER, which perhaps manifested as incipient stress and altered dynamics of the ER. The altered ER sphingolipid flux also affects mitochondrial structure and function that culminates in mitophagy.

*Cert^gt/gt^* cells could have sustained metabolism and viability by activation of autophagic machinery. However, autophagy does not always represent a metabolic end point, rather it is a transient means though which cells attempt to mitigate damage and temporarily maintain homeostasis and quality control. In some instances it can delay cell death and allow them to recover. If the cell stress continues unabated during autophagy, then the cells are diverted into irreversible cell cycle exit (senescence) or are eliminated through programmed cell death (apoptosis) [34]. Trypan blue staining of early and late passage cells showed that the *Cert^gt/gt^* mutant cells don't undergo cell death (data not shown). Autophagy has been shown to be an effector mechanism, which establishes senescence phenotype in the cells. The failure of ceramide transport and subsequent deficiency in the generation of sphingomyelin and complex sphingolipids in the Golgi complex is bound to affect the membrane composition and thereby have deleterious effects on its function. The chronic decline in the functioning of multiple organelle leads to decreased cell viability and results in senescence of the *Cert^gt/gt^* cells.

## Supporting Information

Figure S1 VSVG-GFP transport assay from *Cert^gt/gt^* and *Cert^+/+^* MEFs were performed and images obtained at time points indicated. The co-localization markers used were PDI for ER, GM130 for the Golgi and WGA for the plasma membrane. No difference in the transport of the protein was observed between *Cert^+/+^* and *Cert^gt/gt^* cells.
(JPG)

VideoS1 Late passage (P5) MEfs from *Cert^+/+^* were stained with ER-tracker green and live cell imaging performed over a period of 5 min. The mutant ER is flatter and undergoes more variation in the network over the observed time period compared to the control.
(MOV)

Video S2 Late passage (P5) MEfs from *Cert^gt/gt^* were stained with ER-tracker green and live cell imaging performed over a period of 5 min. The mutant ER is flatter and undergoes more variation in the network over the observed time period compared to the control.
(MOV)

Video S3 **Decreased fission and fusion events in mitochondria from** ***Cert^gt/gt^*** **MEFs.** The mitochondria *Cert^+/+^* were stained and imaged as described under materials and methods. Mitochondrial dynamics of the *Cert^gt/gt^* MEFs is severely compromised.
(MOV)

Video S4 **Decreased fission and fusion events in mitochondria from** ***Cert^gt/gt^*** **MEFs.** The mitochondria *Cert^gt/gt^* were stained and imaged as described under materials and methods. Mitochondrial dynamics of the *Cert^gt/gt^* MEFs is severely compromised.
(MOV)
