# Supplementary figures and images for: Ceramide Transfer Protein Deficiency Compromises Organelle Function and Leads to Senescence in Primary Cells
Source: PLoS One. 2014 Mar 18;9(3):e92142. doi: 10.1371/journal.pone.0092142 (PMC3958450; doi:10.1371/journal.pone.0092142)

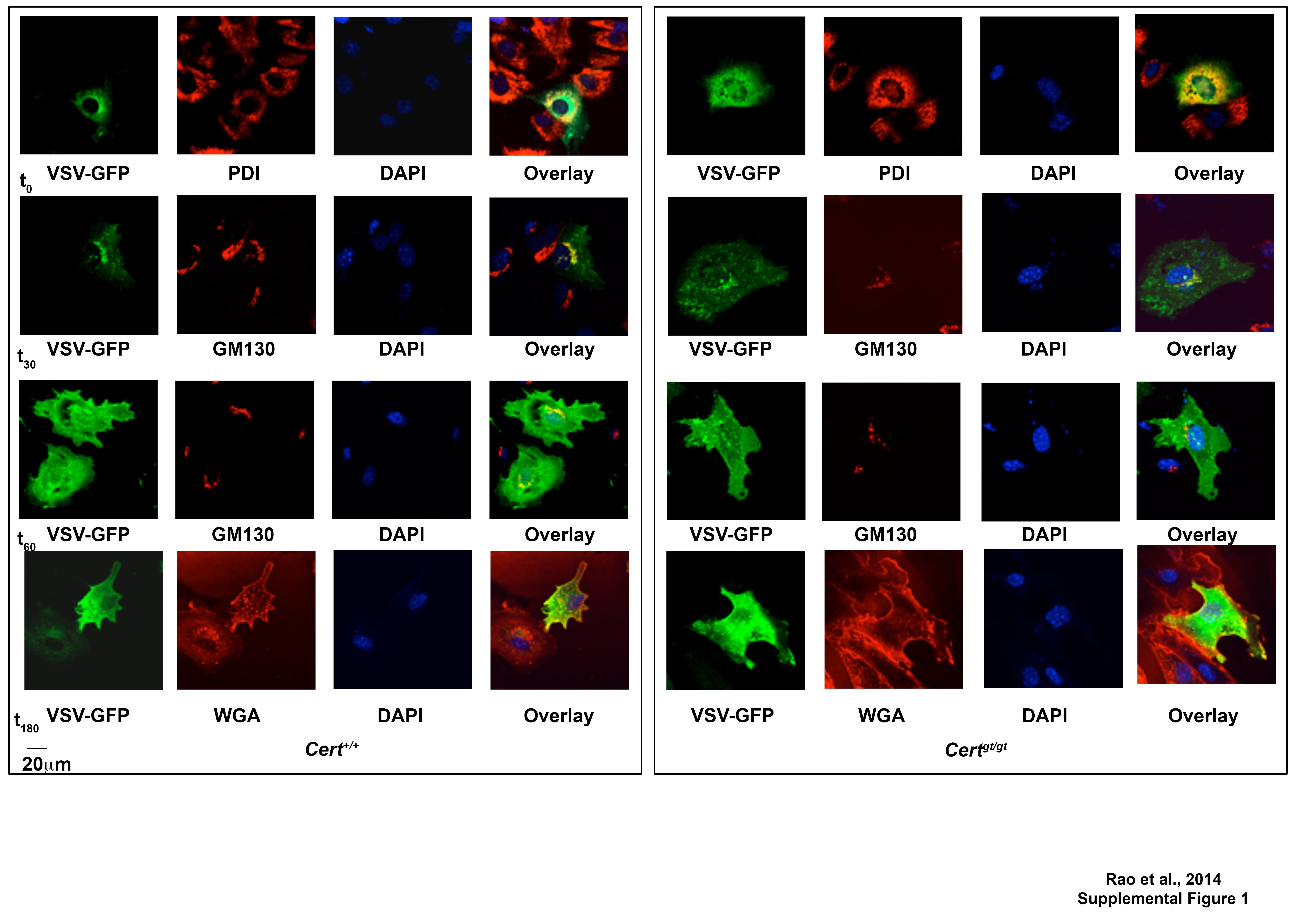

Supplement: Figure S1 — VSVG-GFP transport assay from Certgt/gt and Cert+/+ MEFs were performed and images obtained at time points indicated. The co-localization markers used were PDI for ER, GM130 for the Golgi and WGA for the plasma membrane. No difference in the transport of the protein was observed between Cert+/+ and Certgt/gt cells. (JPG) [file pone.0092142.s001.jpg]
